# A molecular physiological review of vegetative desiccation tolerance in the resurrection plant *Xerophyta viscosa* (Baker)

**DOI:** 10.1007/s00425-015-2320-6

**Published:** 2015-05-22

**Authors:** Jill M. Farrant, Keren Cooper, Amelia Hilgart, Kamal O. Abdalla, Joanne Bentley, Jennifer A. Thomson, Halford J. W. Dace, Nashied Peton, Sagadevan G. Mundree, Mohamed S. Rafudeen

**Affiliations:** Department of Molecular and Cell Biology, University of Cape Town, Private Bag X3, Rondebosch, 7701 South Africa; University of Gadarif, P.O. Box 449, Gadarif, 32211 Al Qadarif Sudan; Queensland University of Technology, PO Box 2434, Brisbane, QLD 4001 Australia

**Keywords:** Physiology, Proteome, Resurrection plant, Transcriptome, Vegetative desiccation tolerance

## Abstract

**Provides a first comprehensive review of integrated physiological and molecular aspects of desiccation tolerance*Xerophyta viscosa*. A synopsis of biotechnological studies being undertaken to improve drought tolerance in maize is given.**

*Xerophyta viscosa* (Baker) is a monocotyledonous resurrection plant from the family Vellociacea that occurs in summer-rainfall areas of South Africa, Lesotho and Swaziland. It inhabits rocky terrain in exposed grasslands and frequently experiences periods of water deficit. Being a resurrection plant it tolerates the loss of 95 % of total cellular water, regaining full metabolic competency within 3 days of rehydration. In this paper, we review some of the molecular and physiological adaptations that occur during various stages of dehydration of *X. viscosa*, these being functionally grouped into early and late responses, which might be relevant to the attainment of desiccation tolerance. During early drying (to 55 % RWC) photosynthesis is shut down, there is increased presence and activity of housekeeping antioxidants and a redirection of metabolism to the increased formation of sucrose and raffinose family oligosaccharides. Other metabolic shifts suggest water replacement in vacuoles proposed to facilitate mechanical stabilization. Some regulatory processes observed include increased presence of a linker histone H1 variant, a Type 2C protein phosphatase, a calmodulin- and an ERD15-like protein. During the late stages of drying (to 10 % RWC) there was increased expression of several proteins involved in signal transduction, and retroelements speculated to be instrumental in gene silencing. There was induction of antioxidants not typically found in desiccation-sensitive systems, classical stress-associated proteins (HSP and LEAs), proteins involved in structural stabilization and those associated with changes in various metabolite pools during drying. Metabolites accumulated in this stage are proposed, inter alia, to facilitate subcellular stabilization by vitrification process which can include glass- and ionic liquid formation.

## Introduction

The vegetative tissues of the majority of plants are highly sensitive to water deficit, losing viability upon loss of between 41 and 70 % (depending on the species) of total water content at full turgor (Höfler et al. 1941). While there are many species common to arid and drought-prone regions (extreme examples being succulents) that are able to resist water deficit in the face of drought, these species are commonly slow growing and few are suitable for consumption by humans or domesticated animals. Cereals, which make up the bulk of current food supplies, being annuals, inherently have poorly developed abilities to resist vegetative water loss and drought-induced crop failure is an increasingly common phenomenon. With climate change models predicting increased desertification in Australia, much of Africa, North and South America and central Europe over the upcoming years (Dai 2013), it is becoming imperative that alternative strategies be developed for crop production under extreme environmental conditions. To date, attempts at producing crops with shortened growing and fruiting/seeding periods, and improved resistance to water deficit stress, have met with only partial success. It is our premise that production of crops with improved water deficit tolerance will be of value for improved food security in the future.

**Fig. 1:**
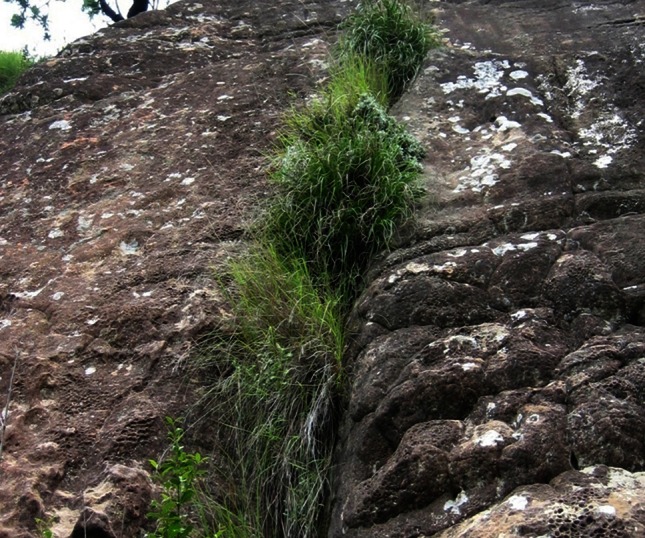
*Xerophyta viscosa* growing chasmophytically in the Cathedral Peak Area of the Drakensburg Mountains, KwaZulu Natal, South Africa

To this end, there are some 135 angiosperm species, commonly referred to as resurrection plants, that have been reported to survive the loss of up to 95 % of total cellular water content, for prolonged periods of time, and which recover full metabolic activity in existing tissues within 24–72 h of rehydration (reviewed inter alia in Gaff 1977; Alpert and Oliver 2002; Farrant et al. 2007; Moore et al. 2009; Dinakar and Bartels 2013; Gaff and Oliver 2013). *Xerophyta viscosa* (Baker) (Fig. 1) is a monocotyledonous resurrection plant that we have used as a model to understand mechanisms associated with vegetative tolerance of extreme water loss (desiccation) with the aim of ultimately utilizing some of these properties in the biotechnological production of more drought-tolerant cereal crops. We have undertaken a top-down, bottom-up systems biology approach to identify key protectants and their regulation during dehydration and recovery from the desiccated state, and are attempting to utilize some of these for the development of drought-tolerant strains of *Zea mays* (maize)—a staple crop in Africa. We present here some of the key findings associated with desiccation tolerance in this species and report briefly on application of these findings in our attempted development of transgenic maize with improved drought tolerance.

## Overview of taxonomy, morphology and distribution

**Fig. 2:**
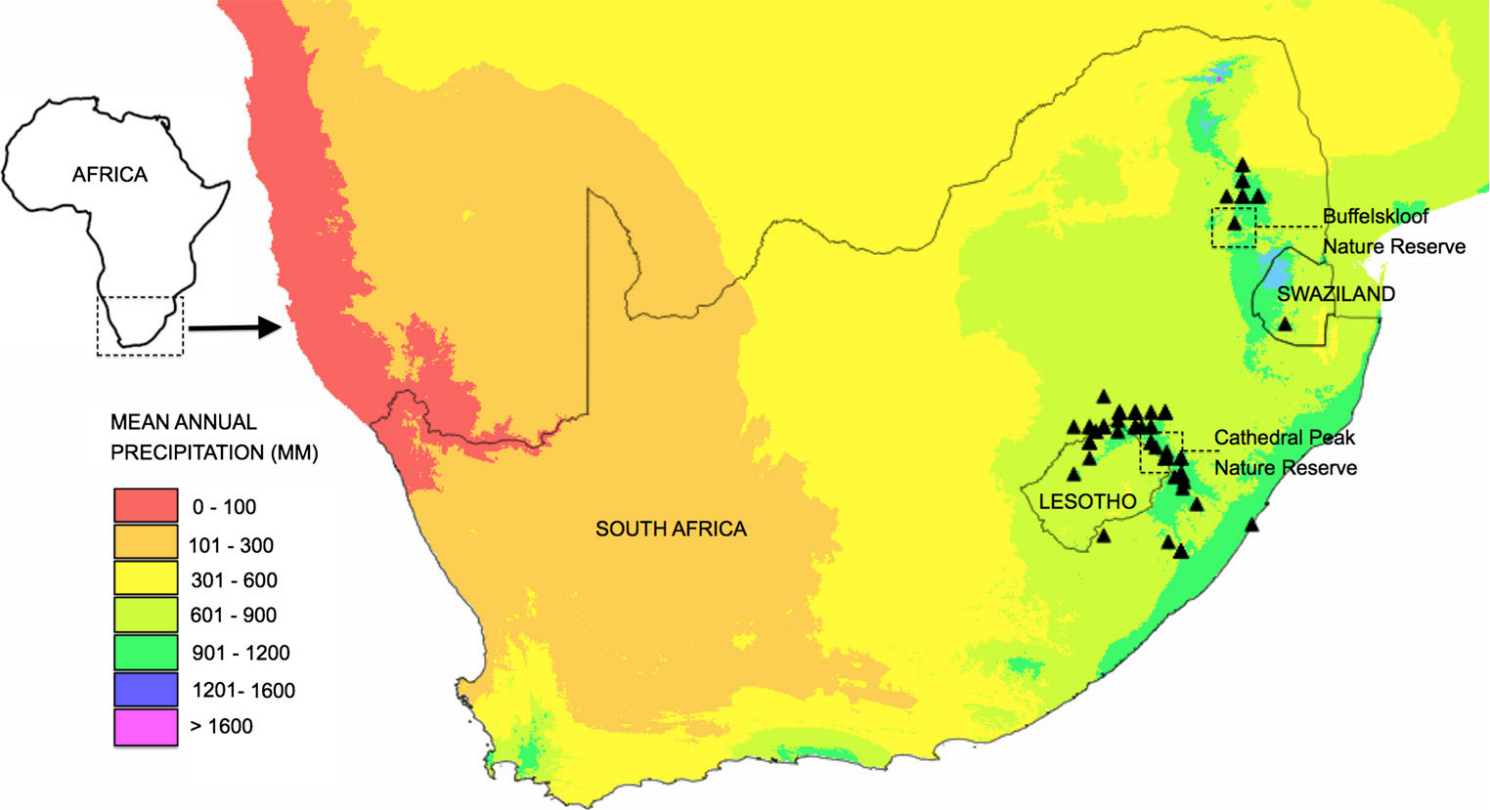
Distribution map of *Xerophyta viscosa* (Baker) in Southern Africa. Mean annual precipitation data for these areas are given. Collection sites are indicated by *dashed squares*

The genus, *Xerophyta* Juss., is a member of the family Velloziaceae and occurs in Africa, Madagascar and the Arabian Peninsula. It is composed of 45 species, at least ten of which are desiccation-tolerant (Coetzee and Schijff 1973; Coetzee 1974; Behnke et al. 2000, 2013; Behnke 2002; Mello-Silva et al. 2011; Gaff and Oliver 2013). *Xerophyta viscosa* Baker occurs in South Africa, Lesotho and Swaziland (Fig. 2) in summer-rainfall Afroalpine, subalpine and coastal grasslands (Mucina and Rutherford 2006), where it inhabits rocky terrain or inselbergs in exposed grasslands (Porembski and Barthlott 2000; Behnke et al. 2013) and is typically found hanging off cliff edges (Fig. 1). Several species of *Xerophyta,* including *X. viscosa,* occur within the species-rich Drakensberg Alpine Centre (DAC), a composite of high-altitude alpine enclaves within the greater Drakensberg range of South Africa renowned for its high plant diversity and endemism (Fig. 2; Carbutt and Edwards 2006). Though mean annual precipitation (MAP) in these regions is moderate (often exceeding 800 mm: Mucina and Rutherford 2006), the harsh chasmophytic habitat of *X. viscosa* leads to frequent periods of severe water deficit, even during the wet season.

**Fig. 3:**
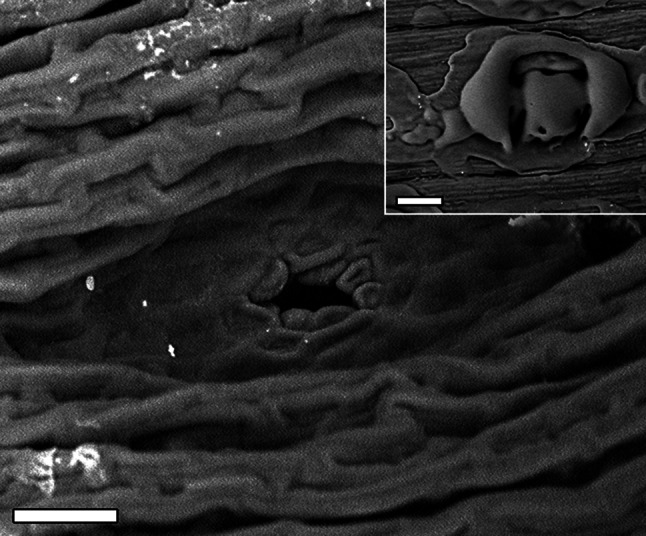
Scanning electron microscope image of the adaxial surface of a hydrated leaf of *X. viscosa* showing a patellar leaf gland. The *inset* shows a gland covered by the sticky exudate that it produces. *Scale bars* represent 20 µm

*X. viscosa* is morphologically distinguished from other desiccation-tolerant *Xerophyta* spp by the formation of chlorophyll-free aquiferous cells between the vascular bundles, the absence of crystals from leaves and the presence of adaxial patellar leaf glands (Fig. 3; Behnke et al. 2013). These glands secrete viscous metabolites including velloziolone, manoyloxide, various diterpenes, phenolic compounds and fatty acids (Naidoo et al. 2009) that play a role in regulating rate of water loss and possibly acting as ‘sunscreens’ to minimize light activation of chlorophyll during the initial stages of drying (Sherwin and Farrant 1998). This process is known to lead to the formation of extensive reactive oxygen species (ROS) which, if not controlled, cause severe subcellular damage and loss of viability (Smirnoff 2008). The presence of elongated, schlerophyllous leaves permit only minimal folding to reduce the surface area exposed to UV radiation upon dehydration, and thus cellular damage is avoided through its poikilochlorophyllous strategy as the photosynthetic apparatus (chloroplasts and chlorophyll) is dismantled upon dehydration (Sherwin and Farrant 1998; Bhatt et al. 2009). In addition, the exposed abaxial surfaces show an accumulation of purple anthocyanin pigment during dehydration which is also thought to act as a ‘sunscreen’, reflecting photosynthetically active radiation during periods prior to complete breakdown of chlorophyll or during resynthesis on rehydration. This resynthesis and the repair of damaged tissues and the resultant energy costs result in longer recovery time as compared to species which retain their photosynthetic apparatus (Sherwin and Farrant 1998).

## Plant collection, maintenance and methodology

Our research has been conducted on plants collected from Buffelskloof Nature Reserve near Lydenberg (Mpumalanga Province, South Africa) and Cathedral Peak Nature Reserve in the Drakensberg mountains (Kwazulu-Natal, South Africa) (indicated in Fig. 2). Plants were maintained under glasshouse conditions at the University of Cape Town (South Africa) until required for dehydration and recovery experiments (Sherwin and Farrant 1996). For such experiments, plants were placed in a controlled environment chamber (16 h light, 350 μmol m^−2^ s^−1^, 25 °C; 8 h dark, 20 °C; 50 % relative humidity) and were allowed to acclimate for a minimum of 2 weeks prior to experimentation. Plant dehydration was achieved by withholding water until tissue water content reached an air dry state (≤5 % water content relative to the fully hydrated state). They were maintained in the dry state for longer than 1 week, following which rehydration was achieved by soil watering. Plants were well watered on the first day and the soil was kept damp for the remainder of the experiments. Tissues were sampled regularly during the drying and rehydration treatments for physiological and molecular experiments referred to below. No significant differences in responses to desiccation were noted among plants collected from these different sites.

**Fig. 4:**
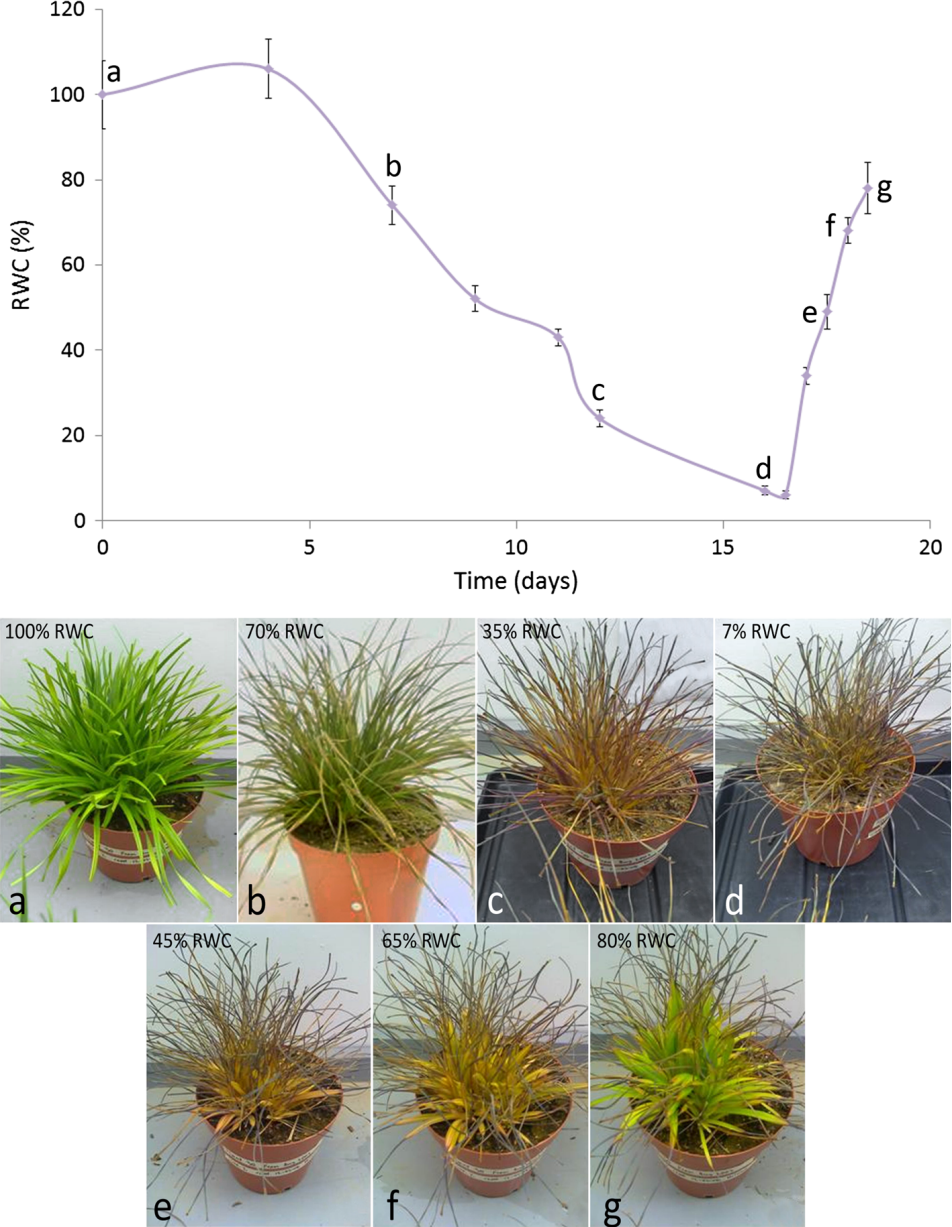
Changes in leaf RWC with images of *Xerophyta viscosa* plants during dehydration (**a**–**f**) and rehydration (**e**–**g**) under a 16 h light, 350 μmol m^−2^ s^−1^, 25 °C and 8 h dark, 20 °C, regime. **a** Fully hydrated plant (100 % RWC). **b** 70 % RWC. **c** 35 % RWC. **d** 7 % RWC. **e** 45 % RWC. **f** 65 % RWC. **g** 80 % RWC. Absolute water contents were determined gravimetrically as described in van der Willigen et al. (2001) with RWC being calculated from this and the mean water content of tissues at full turgor. All the images are of the same plant except image (**b)** which is a plant of similar size and age under the same stress treatment in the same growth room

Dehydration of root and leaf tissues to the air dry state under these conditions takes ca 9 and 15 days, respectively, with leaf rehydration to full turgor taking 3 days (Fig. 4; Sherwin and Farrant 1996; Mundree and Farrant 2000; Kamies et al. 2010). Roots from mature plants of this species are particularly recalcitrant to methodologies typically used for biochemical and molecular studies (Kamies et al. 2010; Kamies 2011) and unless otherwise stated, the data presented below are for leaf tissues only. The changes in leaf water content on drying follow a reverse sigmoidal pattern typical of most resurrection plants we have studied to date (Fig. 4; Farrant et al. 2007), and we have tentatively identified 3 stages to this process. (1) An early response to drying (ERD) in which relative water content (RWC) declines from full turgor to ca 55 % during which leaf colour changes from green to yellow (as seen in Fig. 4a, b) indicative of photosynthetic shutdown (discussed below). (2) A late response to drying (LRD) occurring between 55 and 10 % RWC during which leaves fold adaxially, exposed surfaces becoming anthrocyanin rich (Fig. 4c). (3) Below 10 % RWC, respiration ceases and tissues reach an air dry state (ADS) of ≤5 % RWC (Mundree and Farrant 2000). We have noted in this and other species, that there are subtle quantitative and qualitative changes in transcripts, protein and lipids during maintenance in the ADS (data unpublished), this possibly being equivalent to processes typical of dry after-ripening or dormancy in seeds, in which this metabolism is purported in part to occur in hydrated cellular pockets and is required for germination related processes (Leubner-Metzger 2005: Oracz et al. 2007) and/or due oxidative changes associated with Amadori and Maillard reactions that occur in the dry state (Priestly 1986; Sun and Leopold 1995). To date, there is little known about the cellular and molecular mechanisms involved in these processes and this is an area requiring further investigation.

Stresses associated with water loss (Vertucci and Farrant 1995; Walters et al. 2002; Farrant et al. 2012) during ERD are plasmolysis and cytorrhysis as a consequence of turgor loss, and oxidative as a consequence of ongoing photosynthesis in a water-limited subcellular environment. During the LRD, plant tissues are subject to further oxidative stresses as metabolism becomes increasingly unregulated and metabolic crowding and membrane appression takes place. In the ADS, Maillard and Amadori reactions, autoxidation, protein and membrane destabilization occurs. *X. viscosa* is able to prevent damage associated with such stresses and redirect metabolism for successful recovery upon rehydration as discussed below.

**Fig. 5:**
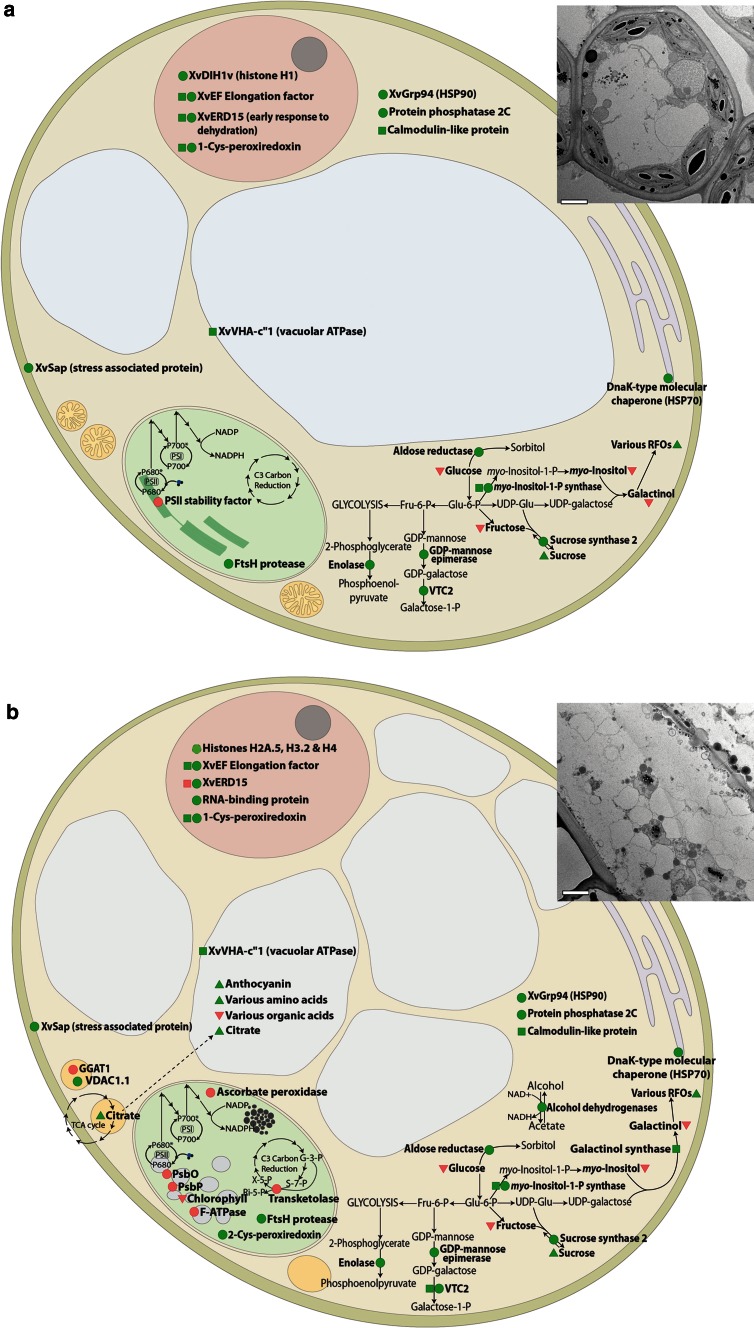
Diagram of changes occurring in cells in early stage dehydration (**a**) and late stage dehydration (**a**) showing the probable locations of these changes. *Green symbols* indicate upregulation and *red symbols* show down-regulation. Proteins are represented by *circles*, transcripts by *squares* and metabolites by *triangles*. *Insets* show transmission electron micrographs at approximately 65 % (**a**) and 37 % (**b**) RWC. *Scale bar* represents 2 µm

Table 1 shows the expression patterns of transcripts and proteins that change significantly during dehydration of *X. viscosa* and putative integrative changes are modelled in Fig. 5. Transcriptome analysis was achieved by using complementation by functional sufficiency in *Escherichia coli* (Mundree and Farrant 2000) and differential screening of dehydration expression libraries (Ndima et al. 2001; Iyer et al. 2008). Proteome analyses were conducted on leaves and isolated nuclei using 2D PAGE and ITRAQ analyses, respectively (Ingle et al. 2007; Abdalla et al. 2010; Abdalla and Rafudeen 2012). There is no genome sequence available for resurrection plants and thus full annotation of transcripts and proteins up- or down-regulated has not been possible. This, together with the challenges of capturing subtle ongoing changes during a prolonged period of dehydration and recovery therefrom, and technological limitations associated with detection of minor but possibly important changes, preclude definitive interpretation of what is actually required for tolerance of extreme water loss. What we describe below can be considered a contribution towards a broader understanding of the phenomenon of vegetative desiccation tolerance.

**Table 1:** List of metabolic components shown to be up- or down-regulated in comparison to fully hydrated tissue

Name	Dehydration stage	Type	Function	Reference
Increased abundance				
Structural: molecular chaperone				
HSP70 (dnaK-type molecular chaperone)	ERD; LRD	P (NP_001048274; GI6746592)	Protein folding, chaperone activity	Ingle et al. (2007); Abdalla and Rafudeen (2012)
Heat shock protein 81–2	LRD*	P (GI9758623)	Protein folding, chaperone activity	Abdalla and Rafudeen (2012)
HSP90 (XvGrp94)	ERD; LRD	P (Q91V38)	Protein folding, chaperone activity	
17.6-kDa class 1 heat shock protein	LRD*	P (GI8671873)	Protein folding, chaperone activity	
XvT6 (Type II LEA-Dehydrin)	LRD*	T (AAP22171)	Protein folding, chaperone activity	Mundree and Farrant (2000)
XvT8 (Type II LEA-Dehydrin)	LRD*	T	Protein folding, chaperone activity	Mundree and Farrant (2000); Ndima et al. (2001)
XvT15 (Lectin)	LRD*	T	Putative reinforcement of the cell wall	Mundree and Farrant (2000)
RHXv (Rehydrin)	LRD*	T	Rehydration associated protein (Oliver et al. 2005)	Mundree and Farrant (2000)
XvSap (stress-associated protein)	ERD; LRD	T, P	Cell membrane-binding protein; maintenance of membrane integrity; putative maintenance of ion homeostasis; putative cell signalling molecule	Garwe et al. (2003)
Structural: other				
GTP-binding proteins	LRD*	P (GI3249104; GI7270538)	Growth, differentiation, cytoskeletal organization	Abdalla and Rafudeen (2012)
Xv5	LRD*	T	Proteolipid subunit of ATPase	Mundree and Farrant (2000)
VDAC1.1 from Lotus corniculatus	LRD	P (AAQ87019)	Voltage-dependent anion channel porins	Ingle et al. (2007)
Histone H4	LRD*	P (GI9758835)	DNA packaging	Abdalla and Rafudeen (2012)
Histone H3.2	LRD*	P (GI145334271)	DNA packaging	Abdalla and Rafudeen (2012)
Putative histone H2A.5	LRD*	P (GI75306451)	DNA packaging	Abdalla and Rafudeen (2012)
XvDIH1v (linker histone H1)	ERD	P	Allows protein contacts for the next level of chromatin structure	Holiday (2007)
Actin putative	LRD*	P (GI30688915)	Cytoskeleton	Abdalla and Rafudeen (2012)
Actin 1	LRD*	P (GI79324605)	Cytoskeleton	Abdalla and Rafudeen (2012)
Oligosaccharyl transferase like	LRD*	P (GI21553942)	Glycolsylation	Abdalla and Rafudeen (2012)
Gene expression: translation				
XvEF Elongation factor (eEF-1α)	ERD; LRD	P, T (At1g07930; T6D22; GI15081765)	Elongation factor during translation	Abdalla and Rafudeen (2012); Conrad (2005)
Translation e-factor eEF-Tu	LRD*	P (GI7268831)	Translation e-factor	Abdalla and Rafudeen (2012)
XvERD15 (early response to dehydration)	ERD; LRD	P, T (AT2G41430)	Poly-A binding protein—translational control; transcript stabilization	Lee (2005)
RNA-binding protein from *Daucus carota*	LRD	P (AAK30205)	Poly-A binding protein—translational control; transcript stabilization	Ingle et al. (2007)
Gene expression: signalling				
Casein kinase α1	LRD*	P (GI79332762)	Protein kinase—stress signalling	Abdalla and Rafudeen (2012)
Malate dehydrogenase	LRD*	P (GI145332399)	Generation of NADPH for ROS-mediated signalling pathways	Abdalla and Rafudeen (2012)
Desiccation-related gene from *Craterostigma plantagineum*	LRD	P (AAA63616)	ABA-responsive: possibly involved in signal transduction	Ingle et al. (2007)
Protein phosphatase Type 2C from *Arabidopsis*	ERD; LRD	P (CAB79642)	Negative regulators of the ABA signalling	Ingle et al. (2007);
Ca-binding protein (calcineurin B-like protein 2)	LRD*	P (GI9758619)	Calcium-binding: signalling	Abdalla and Rafudeen 2012
Patatin-like protein	LRD*	P (GI7270656)	Functions in plant signal transduction as well	Abdalla and Rafudeen (2012)
XvCAM (calmodulin-like protein)	ERD; LRD	T	Calcium-binding: signalling	Conrad (2005)
PAPXv (acid phosphatase homologue)	LRD*	T	Acid phosphatase homologue	Mundree and Farrant (2000)
CBPXv (Ca-binding protein)	LRD*	T	Ca-binding protein	Mundree and Farrant (2000)
Gene expression: gene regulation				
Non-LTR retro element reverse transcriptases	LRD*	P (Q9LGM1)	Modulates and adapts gene regulatory networks during abiotic stress	Abdalla and Rafudeen (2012)
Intron maturases	LRD*	P (Q9BAA0)	Encodes reverse transcriptases (RTs) that function in intron mobility and as maturases to promote RNA splicing	Abdalla and Rafudeen (2012)
Zinc-finger Helicases	LRD*	P (Q8LHZ4)	Contributes to the process of rRNA expression and may function as a transcription factor	Abdalla and Rafudeen (2012)
CDT1 Craterostigma desiccation tolerant protein 1	LRD*	P	Retroelement which functions in gene silencing	Abdalla and Rafudeen (2012)
Functional metabolism				
XvIno1	ERD; LRD	P, T, M	*Myo-*inositol (precursor for important metabolites) synthesis pathway	Lehner et al. (2008);
GolS (galactinol synthetase)	LRD	T	Responsible for the first catalytic step in sucrose and RFO biosynthesis	Peters et al. (2007)
XvALDR4 (aldose reductase)	ERD; LRD	P	Reduction of sugars to alcohols; detoxification of cytotoxic aldehydes (products of lipid peroxidation)	Mundree et al. (2002)
XvVHA-c”1 (vacuolar ATPase gene)	ERD; LRD	T	Creation of pore which assists in adaptation to osmotic pressure fluctuation; housekeeping role to maintain luminal acidification	Marais et al. (2004)
Phosphopyruvate hydratase (enolase) from *Zea mays*	ERD; LRD	P (P26301)	Functions in the formation of phosphoenolpyruvic acid in glycolysis	Ingle et al. 2007
Chloroplast FtsH protease from *A. thaliana*	ERD; LRD	P (CAA68141)	Chloroplast biogenesis; putative PSII repair	Ingle et al. (2007)
Alcohol dehydrogenases	LRD	P (AAY86033; GI9758553)	Ensures NAD+ supply	Ingle et al. (2007); Abdalla and Rafudeen 2012
Putative ferredoxin NADP + reductase	ERD; LRD	P (GI20465661)	Electron transfer during photosynthesis; supply of NADPH for Calvin cycle	Abdalla and Rafudeen (2012)
Sucrose	ERD; LRD	M	Protection; vitrification	Whittaker et al. (2001)
ATP	ERD; LRD	M	Energy metabolism	Whittaker et al. (2001)
ADP	ERD; LRD	M	Energy metabolism	Whittaker et al. (2001)
Dynamin-related protein 1D	LRD*	P (GI68566307)	Plays a role in nucleocytoplasmic transport	Abdalla and Rafudeen (2012)
UDP glucose 4-epimerase	LRD*	P (GI8698725)	Biosynthesis of UDP-Gal, a precursor for several important metabolites	Abdalla and Rafudeen (2012)
F28C11.12 ADP-ribosylation factor	LRD*	P (GI8778579)	Vesicle transport; may facilitate overexpression of sucrose	Abdalla and Rafudeen (2012)
XvSuS2 (Sucrose synthase type 2)	ERD	P	Synthesis (and breakdown) of sucrose	Ngwarai (2014)
ATP-binding protein	LRD*	P (GI145334803)	Cellular transport	Abdalla and Rafudeen (2012)
Raffinose	ERD	M	Protection; water replacement? Vitrification?	Peters et al. (2007)
Stachyose	ERD	M	Protection; water replacement? Vitrification?	Peters et al. (2007)
Verbascose	ERD	M	Protection; water replacement? Vitrification?	Peters et al. (2007)
Anthocyanin	LRD	M	Protection from oxidative stress	Mundree and Farrant (2000);
Aspartic acid	LRD	M	Free amino acid pool; vitrification?	Dace (2014)
Glycine	LRD	M	Free amino acid pool; vitrification?	Dace (2014)
Phenylalanine	LRD	M	Free amino acid pool; vitrification?	Dace (2014)
Threonine	LRD	M	Free amino acid pool; vitrification?	Dace (2014)
Tryptophan	LRD	M	Free amino acid pool; vitrification?	Dace (2014)
Tyrosine	LRD	M	Free amino acid pool; vitrification?	Dace (2014)
Citric acid	LRD	M	Part of TCA acid cycle; vitrification?	Dace (2014)
Antioxidant metabolism				
1-Cys XvPer 1 (peroxiredoxin type I)	ERD; LRD	P, T (GI8778528)	Enhance plant ability to cope with ROS exacerbated by abiotic stresses	Mowla et al. (2002); Abdalla and Rafudeen (2012)
2-Cys peroxiredoxin from *O. sativa*	LRD	P	Enhance plant ability to cope with ROS exacerbated by abiotic stresses	Ingle et al. (2007); Govender (2006)
XvVTC2	ERD; LRD	P	Part of ascorbate metabolism	Bresler (2010)
XvVTC2	LRD	T	Part of ascorbate metabolism	Bresler (2010)
XvGME (GDP-mannose-3′,5′-epimerase from *O. sativa*)	ERD; LRD	P (Q2R1V8)	Part of ascorbate metabolism	Ingle et al. (2007); Abdalla and Rafudeen (2012)
NADPH:protochloro-phyllide O. reductase A	LRD*	P (GI968975)	Functions in antioxidant defense and protection against ROS via energy transfer	Abdalla and Rafudeen (2012)
NADPH:proto-chlorophyllide oxido reductase B	LRD*	P (GI968977)	Functions in antioxidant defense and protection against ROS via energy transfer	Abdalla and Rafudeen (2012)
Decreased abundance				
Structural: other				
PSII stability factor HCF136 from *Oryza sativa*	ERD	P (BAD62115)	Photosynthetic machinery	Ingle et al. (2007)
PsbO from *A. thaliana*	LRD	P (CAA36675)	Photosynthetic machinery	Ingle et al. (2007)
PsbP from *Xerophyta humilis*	LRD	P (AAN77240)	Photosynthetic machinery	Ingle et al. (2007)
Putative S-phase-specific ribosomal protein	LRD*	P (GI7270417)	Protein synthesis	Abdalla and Rafudeen (2012)
30S ribosomal protein S5	LRD*	P (GI75101015)	Protein synthesis	Abdalla and Rafudeen (2012)
Ribosomal protein 5B	LRD*	P (GI79324564)	Protein synthesis	Abdalla and Rafudeen (2012)
40S ribosomal protein	LRD*	P (GI464720)	Protein synthesis	Abdalla and Rafudeen (2012)
40S ribosomal protein S19-like	LRD*	P (GI21555157)	Protein synthesis	Abdalla and Rafudeen (2012)
Cytochrome b559	LRD*	P (GI114152861)	Electron transport	Abdalla and Rafudeen (2012)
Functional metabolism				
Transketolase from *Solanum tuberosum*	LRD	P (CAA90427)	Pentose phosphate pathway and Calvin cycle	Ingle et al. (2007)
F-ATPase (a subunit) from *Ranunculus macranthus*	LRD	P (AAZ03784)	Energy metabolism	Ingle et al. (2007)
Glu:glyoxylate aminotransferase I from *Arabidopsis thaliana*	LRD	P (AAN62332)	Photorespiration	Ingle et al. (2007)
T1N15.8; probable glutamine synthetase	LRD*	P (GI8778687)	Nitrogen metabolism	Abdalla and Rafudeen (2012)
Putative glutamine synthetase	LRD*	P (GI28393681)	Nitrogen metabolism	Abdalla and Rafudeen (2012)
NADP-specific isocitrate dehydrogenase	LRD*	P (GI6227018)	TCA cycle	Abdalla and Rafudeen (2012)
Glycolate oxidase	LRD*	P (GI62320779)	Recycling of 2-phosphoglycolate produced from Calvin cycle; Putative role in ROS-mediated cell signalling	Abdalla and Rafudeen (2012)
Glucose	ERD	M	Energy metabolism	Peters et al. (2007)
Fructose	ERD	M	Energy metabolism	Peters et al. (2007)
*Myo-*Inositol	ERD	M	Energy metabolism	Peters et al. (2007)
Galactinol	ERD	M	Energy metabolism	Peters et al. (2007)
Chlorophyll	LRD	M	Photosynthesis	Mundree and Farrant (2000);
Malic acid	LRD	M	Metabolism	Dace (2014)
Succinic acid	LRD	M	Metabolism	Dace (2014)
Antioxidant metabolism				
Ascorbate peroxidase from *A. thaliana*	LRD	P (CAA66925)	Antioxidant	Ingle et al. (2007)
Catalase	LRD*	P (GI7270460)	Antioxidant	Abdalla and Rafudeen (2012)

Accession numbers given where available

*P* protein, *T* transcript, *M* metabolite, *ERD* early stage dehydration, *LRD* late stage dehydration, LRD* tissue from late stage dehydration only analysed, early stage tissue untested

## Early stage dehydration

### Gene expression (signalling and translation)

The initiation of the response to water deficit begins with signal transduction. However, changes in gene expression via these signalling events require and depend upon prior post-translational modifications to the nucleosome histone structure and DNA methylation (Chinnusamy and Zhu 2009; Sahu et al. 2013). The chromatin is remodelled under water deficit stress conditions by various proteins such as histone modification enzymes and linker histone H1 among others (Kim et al. 2010). The latter specifically allows protein contacts for the next level of chromatin structure and are easily modified post-translationally. A few drought-induced linker histone H1 variants have been characterized to date and seem not to be essential for growth and development (Wei and O’Connell 1996; Ascenzi and Gantt 1997). It has been suggested that these histone H1 variants are required to change the transcriptional activity of the cell undergoing water loss by altering the chromatin structure to either allow and/or suppress the binding of transcription factors to the DNA (Scippa et al. 2000). Transcript levels of a *X. viscosa* linker histone H1 variant, XvDIH1v, homologous to the drought-induced H1 variants, increases when water content declines to 55 % RWC, after which it appears to be down-regulated (Holiday 2007). Overexpression of this protein in tobacco increases tolerance to various abiotic stresses (Wang et al. 2014).

Our studies also show an early upregulation (at 75 % RWC) of a protein phosphatase [homologous to Type 2C from *A. thaliana* (CAB79642)] at both the protein and transcript level, the protein declining upon further dehydration (Ingle et al. 2007; Umezawa et al. 2009). Phosphatases are thought to act as negative regulators of protein kinase and ABA signalling pathways and such a negative feedback loop allows the plant to reset the ABA signalling pathway in order to continually monitor the presence or absence of ABA (Gosti et al. 1999). Interestingly, an ABA-responsive desiccation-related protein is synthesized de novo in the LRD, which has been proposed to be related to regulation of signalling (Ingle et al. 2007).

Transcripts of a calcium-binding protein containing three EF-hands with high similarity to plant calmodulins (Xv-CAM) increased significantly upon initial drying, with the protein itself becoming evident (through use of western blot analysis) below water contents of 55 % (Conrad 2005; Abdalla and Rafudeen 2012). The protein remains highly expressed until the early stages of rehydration (40 % RWC) after which little protein was detected (Conrad 2005). Ca^2+^-mediated signalling in response to abiotic stresses is a well reported phenomenon and overexpression of rice calmodulin (*Os MSR2*) enables enhanced drought tolerance in *Arabidopsis thaliana* (Xu et al. 2011). While the molecular and physiological functions of such proteins are not completely understood, it is widely thought that they regulate calcium levels through a tight networks of sensory proteins, membrane pumps and ion channels, which inter alia allow for the spatial and temporal management of the resultant calcium signal with consequent down-stream ABA signalling and protectant effects (McAinsh and Hetherington 1998; Pittman and Hirschi 2003; Hirschi 2004).

In *A. thaliana* a number of proteins are induced as an early response to drying and which have thus been termed ERD proteins (Kiyosue et al. 1994) Transcripts and proteins of an ERD15-like protein are strongly induced at 75 % RWC, with expression being maintained until ca 30 % RWC, after which both protein and transcripts decline, being absent during the early stages of rehydration (Lee 2005). XvERD15 is a small, acidic protein with 46, 41 and 38 % overall amino acid identity to ERD15 homologues in rice, tomato and Arabidopsis, respectively. Kariola et al. (2006) showed using *A. thaliana* lines in which AtERD15 was either silenced or over-expressed, respectively, and by measuring *AtERD15* transcript content in *abi* mutants, that ERD15 is a negative regulator of the ABA response. It was proposed that the modulation of the amounts of ERD15 synthesized alters the responsiveness to the ABA signal, possibly downstream of ABI 1 and ABI 2, via regulating stomatal aperture (Kariola et al. 2006; Aalto et al. 2012). Those authors further proposed that ERD15 acts to delay the stress signal (abiotic or biotic, respectively) until sufficient stimuli are received before the plant commits to the large-scale adaptation to the specific stress. It is speculated that XvERD15 provides an ABA regulated threshold, or ‘tipping point’, for commitment to mechanisms that ensure tolerance of extreme water loss.

### Responses to mechanical stress

Reduction in cell volume as a consequence of water deficit results in mechanical stresses of plasmolysis as tension is placed on regions of the plasma membrane attached to cell walls and ultimately cytorrhysis, during which walls collapse and cell death ensues (Iljin 1957). In *X. viscosa* leaf tissues, these stresses appear to be ameliorated largely by the sequential subdivision of the large central vacuole into smaller vacuoles in which water is replaced with compatible solutes (Mundree and Farrant 2000), the result of which becomes clearly evident at water contents below 55 % RWC (insets, Fig. 5). In parallel with these changes, there was is upregulation of both transcripts and proteins of a vacuolar adenosine triphosphatase (V-ATPase) proteolipid subunit c″homologue (XvVHA-c″1) which is proposed to be involved, inter alia, in energy required for vacuolar transport (Marais et al. 2004).

Unlike in many other resurrection plants such as *Craterostigma* spp. (Vicré et al. 1999, 2004) and *Boea hygroscopica* (Wang et al. 2009), there is little evidence of wall folding during drying. Wall composition analysis, involving assessment of monosaccharide composition, comprehensive microarray polymer profiling and FT-IR spectroscopy in combination with multivariate data analysis, has confirmed a lack of quantitative and architectural changes during dehydration (Moore et al. 2013). However, walls of this species contain high amounts of arabinosylated xylans and arabinogalactan proteins (AGPs). Elevated arabinan polymers (as arabino pectins typified in dicotyledons or arabinosylated xylans in monocots) in combination with AGPs are a common feature in all resurrection plants (7 species) subject to such analysis (Moore et al. 2013). These authors have proposed that, since arabinose polymers are highly mobile, they allow wall flexibility (Foster and Ablett 1996; Renard and Jarvis 1999) and have a high water-absorbing capacity (Goldberg et al. 1989; Belton 1997) which is important for rehydration. Such a constitutively high arabinan content is indicative of a constant preparedness for dehydration-rehydration.

### Oxidative stress and antioxidants

Water deficit results in oxidative stresses as a consequence of perturbation of metabolism in general, but metabolism involving electron transport is particularly susceptible to ROS formation (Foyer et al. 1994; Halliwell and Gutteridge 1999; Apel and Hirt 2004; Foyer 2010). Electron leakage during photosynthetic electron transport and the formation of singlet oxygen are significantly increased when cells of photosynthetic tissues suffer water loss and this has frequently been cited as a primary cause of damage and resultant plant death in most species (Seel et al. 1992; Smirnoff 1993; Kranner and Birtić 2005). *X. viscosa* is a poikilochlorophyllous resurrection plant (Sherwin and Farrant 1998) in which chlorophyll is degraded and thylakoids dismantled, effectively shutting down photosynthesis by 55 % RWC, while simultaneously reducing photosynthetically produced ROS (Mundree and Farrant 2000). Chlorophyll degradation is a regulated process (and not simply a consequence of photooxidative breakdown), occurring via the pheophorbide α oxygenase (PAO) phyllobilin pathway, the phyllobilin catabolites formed being accumulated in the vacuole and subsequently degraded during early rehydration (Christ et al. 2013). Dismantling of thylakoid membranes is typified by an early onset of decreased abundance of LHCb1, chlorophyll-binding light harvesting protein of PSII, and the thylakoid luminal protein, HCF136 required for PSII stability. These data support the contention that controlled shutdown of particularly PSII complexes during desiccation plays a considerable role in minimizing dehydration induced photosynthetic ROS (Dinakar et al. 2012; Farrant et al. 2012; Christ et al. 2013). Interestingly, this is true too of the homoiochlorophyllous resurrection plant *Craterostigma pumulim*, in which photosynthetic shutdown has been shown to be achieved primarily by structural rearrangements of PSII complexes into a photochemically quenched state (Charuvi et al. 2015). Reappearance of chlorophyll and thylakoid proteins is initiated after 24 h of rehydration (50 % RWC) and the photosynthetic apparatus is fully reconstituted after 3 days, this corresponding to achievement of full turgor (Sherwin and Farrant 1996; Christ et al. 2013; also shown in Fig. 3). In the related resurrection plant, *Xerophyta humilis*, resynthesis of chlorophyll and initial reconstitution of thylakoid membranes is independent of de novo transcription, suggesting that stable storage of transcripts is required for re-activation of photosynthesis during recovery (Dace et al. 1998). This is likely to occur in *X. viscosa* as well, as there is increased presence of RNA binding proteins purported to stabilize transcripts during the late stages of drying (Ingle et al. 2007; Abdalla and Rafudeen 2012).

During the ERD in *X. viscosa*, there is an increase in ascorbate and GDP mannose-3′, 5′-epimerase (an enzyme involved in ascorbate synthesis) as well as the activities of glutathione reductase (GR), chloroplastic superoxide dismutase (CuZn SOD), catalase and ascorbate peroxidase (Sherwin and Farrant 1998; Mundree and Farrant 2000; Farrant et al. 2007; Ingle et al. 2007; Kamies et al. 2010). Early induction of ‘housekeeping’ antioxidant potential occurs in desiccation-sensitive species in response to water deficit (reviewed in Farrant et al. 2007) and thus *X. viscosa* behaves no differently. However, it is the maintenance of this and other antioxidant potential during the later stages of drying (reviewed below) that help differentiate this species from drought-sensitive plants.

While numerous metabolic shifts are likely to occur during ERD, the major differences observed in our studies are portrayed in Fig. 5a. In particular, there is an increase in metabolism associated with the ascorbate pathway and redirection of metabolism to the increased formation of sucrose, raffinose family oligosaccharides (RFOs) and sorbitol. Accompanying these there is evidence of increased glycolysis resulting inter alia in increased carbon allocation to the phosphophenol pyruvate pathway.

## Late stage dehydration

Dehydration below 55 % (Fig. 4c–e), a relative water content beyond which most plant species lose viability, is accompanied by numerous shifts in the transcriptome, proteome and metabolome (Table 1; Fig. 5b).

### Gene expression (signalling and translation)

In a study of the nuclear proteome during drying of *X. viscosa*, Abdalla and Rafudeen (2012) have shown considerable upregulation of proteins involved in signalling processes that they propose are relevant predominantly in the late stages of dehydration, in that such proteins were highly expressed at 35 % RWC and below. Among these are a Ca-binding protein with similarity to calcineurin B-like protein 2, a malate dehydrogenase that is involved in the generation of NADPH for ROS-mediated signalling reactions (Apel and Hirt 2004), patatins and glycoproteins that function in signal transduction. Furthermore, the cell signalling-related protein phosphatase Type 2C, which first appear in the early stages of dehydration, is maintained at high concentrations during LRD. Such changes have been implicated in the regulation of ROS-scavenging machinery and mediation of other cell signalling processes required under abiotic stress situations (Davis 2005).

Other genes noted to be upregulated in *X. viscosa* during late stage dehydration include retroelements (speculated to be instrumental in gene silencing, particularly in response to stress, Nakaminami et al. 2012), XvZPR1 (a zinc-finger helicase and a proposed transcription factor) and expression of an RNA-binding protein homologous to a maturase found in *Daucus carota* which is ostensibly involved in binding and stabilization of mRNA (Wang et al. 2014). Similar changes occur during the final stages of dehydration in the closely related *X. humilis* (Collett et al. 2003, 2004), giving support to the contention that they are highly relevant to overall stabilization of the subcellular milieu at low water contents and maintenance of quiescence while in the dry state. In addition, there is an upregulation of an oligosaccharyl transferase-like protein that likely functions in post-translational modification by *N*-glycosylation (Abdalla and Rafudeen 2012) and a membrane-binding protein XvSAP1 (a stress-associated protein with homologies to a WCOR413, a cold-responsive protein from wheat, rice and *A. thaliana* and a potassium transporter) that might play a role in signal transduction in response to abiotic stresses (Garwe 2003; Garwe et al. 2003; Iyer et al. 2008).

### Oxidative stress and antioxidants

During the LRD there is further decline in proteins associated with photosynthesis (particularly psbP and components of the luminal oxygen evolving complex (OEC) of PSII (Ingle et al. 2008) further minimizing potential ROS generation from photosynthetic activity. However, drying below 55 % RWC results in ROS formation from ongoing respiration and perturbation of other metabolisms as a consequence of the considerably reduced aqueous environment within tissues (Mundree and Farrant 2000; Walters et al. 2002). In general, protein housekeeping antioxidants do not show a further increase in activity during LRD, and some, like catalase and ascorbate peroxidase decline in activity at this stage. Interestingly, however, enzyme antioxidants that are present remain un-denatured during drying and retain the ability to detoxify ROS during the LRD and early rehydration, as evidenced by in vitro analysis of extracted proteins (Sherwin and Farrant 1996; Mundree and Farrant 2000; Farrant et al. 2007). Transcripts of GDP-l-galactose phosphorylase (Xv VTC2; the first committed enzyme in the synthesis of ascorbate) increase by 1000-fold during LRD, but protein expression and ascorbate concentrations remain un-elevated until the early stages of rehydration after which considerable increases in both occur (Bresler 2010). These data collectively suggest that maintenance (however this is achieved) of housekeeping antioxidant potential during drying and early rehydration is important to survival of extreme water loss. But it is clear that other antioxidant systems, not usually upregulated in desiccation-sensitive material, are also required for survival of desiccation. For example, there is significant upregulation of a nucleus-associated antioxidant 1-Cys peroxiredoxin (XvPer1, Mowla et al. 2002), to date reported to be specific to desiccation-tolerant seed tissues (Leprince and Buitink 2010). There is also an increase in transcript and protein of a type II peroxiredoxin (*XvPrx2*), a chloroplast-targeted protein that reduces peroxide substrates to the corresponding alcohol and water (Govender 2006; Dietz 2011). Increased polyphenol content during late drying have also been implicated in antioxidant defence as their relative antioxidant potential as determined by FRAP (ferric reducing/antioxidant power) and DPPH (2,2-diphenyl-1-picrylhydrazyl) shows higher activity in *X. viscosa* (and other resurrection plants) than in related desiccation-sensitive species (Farrant et al. 2007, 2012). Given the complexity involved in redox balancing (Foyer and Noctor 2005, 2009) it is probable that these are only a few of the components involved in antioxidant metabolism during LRD, with cytoplasmic vitrification, which progressively occurs during this stage (see below), further contributing to ROS stasis.

### Macromolecular stabilization and induction of metabolic quiescence

During the late stages of drying cellular contents become concentrated, increasing the likelihood of inappropriate molecular interactions and membrane appression. Ultimately the lack of sufficient water to surround macromolecules causes their denaturation and loss of membrane integrity (Vertucci and Farrant 1995; Walters et al. 2002). The ability to withstand such water loss therefore requires unique protective adaptations.

It is thus no surprise that during LRD there is induction of four heat shock proteins (HSP 70, 81–2; 90 and a 17.6-kDa class 1 HSP) with putative chaperonin activity (Walford et al. 2003; Ingle et al. 2007; Abdalla et al. 2010; Abdalla and Rafudeen 2012). HSPs accumulate during acquisition of desiccation tolerance in seeds (Vierling 1991; Wehmeyer et al. 1996; Buitink et al. 2006) and other resurrection plants (Alamillo et al. 1995; Walford 2008), firmly establishing their role in desiccation tolerance. In addition to the classical protein folding role of chaperonins, they (XvHSP 90 in particular) have been evoked in binding to non-native proteins preventing their aggregation (Walford et al. 2003) and in some instances in signal transduction via interactions with plant growth regulators and certain protein kinases (Picard et al. 1990; Bohen and Yamamoto 1994; Buchner 1999).

Although precise functions of most Late Embryogenesis abundant (LEA) proteins are still largely unknown, many have been implicated in tolerance of water deficit stress (reviewed in Cuming 1999; Illing et al. 2005; Leprince and Buitink 2010; Tunnacliffe and Wise 2007; Tunnacliffe et al. 2010; Farrant et al. 2012). Unpublished data from our laboratory indicate the presence of 21 LEA-like proteins in the genome of *X. viscosa*, only two of which (XvT6 and XvT8, both Type II or dehydrin-like proteins) have been functionally characterized (Mundree and Farrant 2000; Ndima et al. 2001). Transcripts and proteins are ABA inducible and become evident upon drying below 40 % RWC, expression declining rapidly during rehydration in a pattern typical of desiccation-tolerant plant tissues (Illing et al. 2005; Leprince and Buitink 2010). Dehydrins are induced in response to water deficit in several desiccation-tolerant systems (Ingram and Bartels 1996; Close 1997) and are constitutively expressed in the moss *Tortula ruralis* (Bewley et al. 1993). Because such proteins are mostly unfolded in aqueous solutions, it is experimentally difficult to assign to them a structure and function and thus the predicted roles for LEA proteins have been based largely on RNA sequence information. These include (1) water replacement molecules and/or hydration buffers; (2) ion sequesters; (3) chaperonins and/or heat shields; (4) protein/membrane anti-aggregants and membrane stabilizers; and (5) promoters of vitrification (Bray 1997; Hoekstra et al. 2001; Wise and Tunnacliffe 2004; Bartels 2005; Goyal et al. 2005; Mtwisha et al. 2006; Berjak et al. 2007; Chakrabortee et al. 2007; Tunnacliffe and Wise 2007; Farrant et al. 2012). All these functions can be visualized to be of relevance to maintenance of subcellular structural integrity in water-deprived environments and indeed in the ultimate promotion of metabolic quiescence in the dry state. Interestingly, there is also transcriptional upregulation of a rehydrin protein (RHXv) (Mundree and Farrant 2000), induced in response to rehydration of the desiccation-tolerant moss, *Tortula ruralis* (Oliver et al. 2005) and it is proposed that it has a dehydrin-like function, potentially protecting membranes and/or facilitating lipid transport for reconstitution of damaged membranes during rehydration.

Other gene products upregulated during LRD and which may play a role in maintenance of structural integrity during desiccation are XvSAP1, actin, several histones, GTP-binding proteins and oligosaccharide transferases (Mundree and Farrant 2000; Ingle et al. 2007; Abdalla and Rafudeen 2012). XvSAP1 in addition to being a putative signalling molecule (reviewed above) has been proposed by Garwe (2003) and Garwe et al. 2003 to be involved during the late stages of drying in ion homeostasis and membrane stabilization. Actin participates in more protein–protein interactions than any known protein and its ability to transition between monomeric (G-actin) and filamentous (F-actin) states under the control of nucleotide hydrolysis, ions, and a large number of actin-binding proteins, make it a critical component of many cellular functions, including maintenance of cell shape and polarity and the regulation of transcription (Dominguez and Holmes 2011). The precise role of increased actin during water deficit is unknown, but increased levels at low water contents, when transcription and translation processes are reduced, might suggest that along with increased GTP-binding proteins, actin is involved in processes associated with structural stabilization in the dry state. Increased expression of histone proteins (H2A.5, H3.2 and H4) is speculated to be involved in regulation of late gene expression and in protection and stabilization of DNA in the dry state (Abdalla and Rafudeen 2012). Oligosaccharide transferases serve as glycosylating agents and as such could play a number of roles during late stage dehydration, including stabilization of biopolymers (DNA, RNA and proteins) and/or in co- and post-translational modifications that facilitate tolerance of water deficits (Varki and Lowe 2009).

### Metabolite changes

A common feature in plant desiccation tolerance is the accumulation during the late stages of drying of high amounts of sucrose and RFOs (reviewed in Berjak et al. 2007; Bartels and Hussain 2011; Farrant et al. 2012; Dinakar and Bartels 2013, Elsayed et al. 2014). In *X. viscosa* there is a 30- to 50-fold increase sucrose, a 25-fold increase in raffinose and stachyose and a tenfold increase in verbascose content, along with depletion of monosaccharides (glc and fru), galactinol and *myo*-inositol of leaves during the LRD (Whittaker et al. 2001; Peters et al. 2007; Farrant et al. 2007). Accompanying these changes there is an increase in transcript and protein levels of an isoform of sucrose synthase (XvSUS2) (Ngwarai 2014) and increased hexokinase (Whittaker et al. 2001), galactinol synthase (GolS; Peters et al. 2007) and *myo*-inositol 1-phosphate synthase (Lehner et al. 2008) activities in *X. viscosa* leaves during drying. We propose that accumulation of these particular sugars facilitate the formation non-crystalline glasses (Leopold and Vertucci 1986; Vertucci and Farrant 1995; Buitink et al. 2002; Berjak et al. 2007) while at the same time removing reducing sugars and aldoses that, in high quantities under water deficit conditions, can lead to formation of ROS via Maillard reactions (Walters et al. 2002). Other proposed roles for selective oligosaccharide accumulation during extreme water loss are replacement of water by substitution of H bonds lost during dehydration (Crowe and Crowe 1986; Crowe et al. 1989), as antioxidants (Van den Ende and Valluru 2009) and as sugar-sensors capable of regulating of gene expression under conditions of oxidative stress (reviewed in Rosa et al. 2009). Furthermore, oligosaccharides accumulated during drying are rapidly mobilized during rehydration, probably serving as an energy source for repair and recovery.

In addition to the reallocation of soluble sugars, a number of other primary metabolites are regulated in response to desiccation in the closely related *Xerophyta humilis* (Dace 2014), with preliminary data suggesting that *X. viscosa* behaves similarly. Specifically, the amino acids aspartic acid, glycine, phenylalanine, threonine, tryptophan and tyrosine increase in abundance during LRD. This is likely to be a consequence of protein degradation, resulting in pools of free amino acids necessary for early recovery. The pools of simple organic acids change substantially, with citric acid increasing while malic and succinic acids decreasing in abundance (Dace 2014). The change in organic acid profiles may contribute to the formation of ionic liquids that have been proposed to facilitate and maintain solubility of macromolecules in the absence of water (Choi et al. 2011).

The overall changes observed during late stages of dehydration are summarized in Fig. 5b. These changes are likely to not only facilitate tolerance of extreme water deficit, but also in preparation for reconstitution of metabolism on rehydration. Within the nucleus there is upregulation of histone-like proteins proposed to stabilize the DNA in the dry state and RNA-binding proteins that stabilize transcripts required for rehydration. Among these are likely to be transcripts required for regeneration of the photosynthetic machinery which is disassembled during drying. Interestingly, there is upregulation of an FtsH protease that has been purported to be involved in chloroplast biogenesis and photosystem II repair (Zaltsman et al. 2005; Ingle et al. 2007). Key metabolic changes occurring during LRD involve further shifts in carbon allocation towards specific primary metabolic pathways, including the synthesis of sucrose, RFOs, citric acid and various amino acids, with concomitant changes in enzymes associated with their anabolism. Many of these are probably accumulated in the numerous small vacuoles (inset Fig. 5b) facilitating mechanical stabilization in the dry state. Furthermore, such metabolites have been implicated in stabilization of the subcellular milieu by vitrification which can include glass- and ionic liquid formation.

## Biotechnological studies

One of the aims of our research on *X. viscosa* is the development of crops with improved drought tolerance. As it is a monocot it could serve as a model for understanding what might be required for the production of drought-tolerant cereals. As outlined above, desiccation tolerance is a complex phenomenon involving the regulated expression of numerous genes, the products of which interact through chemical and physical processes to limit damage associated with water deficit and to facilitate survival in the dehydrated state. Most resurrection plants have a large genome and thus are difficult to transform, precluding genetic studies that might facilitate understanding of key regulators of desiccation tolerance. While it is unlikely that transformation of drought-sensitive species with only one or two genes will actually result in complete tolerance of water loss, such approaches have met with some improvement in drought tolerance (e.g. Xu et al. 2011; reviewed in Deikman et al. 2012). We are thus attempting to transform maize with some of the genes shown to be highly upregulated during drying in *X. viscosa.* The importance of the development of drought-tolerant maize for Africa is exemplified by the Water Efficient Maize for Africa (WEMA) project which is managed by the African Agricultural Technology Foundation (AATF; Oikeh et al. 2014).

We have shown previously that selected *X. viscosa* genes transformed into dicots such as *A. thaliana* and tobacco confers abiotic stress tolerance to these transgenic plants when exposed to a variety of abiotic stresses (Garwe et al. 2006; Govender 2006; Mundree et al. 2006; Maredza 2007; Kumar et al. 2013). Furthermore, we have used the stress-inducible promoter of *XvSap* to drive the expression of reporter genes in *A. thaliana*, tobacco and maize tissue culture (Black Mexican sweetcorn) (Oduor 2009; Ellick 2012). The *XvSap* promoter is only induced upon abiotic stress treatments and could be useful in the future improvement of drought tolerance of crop plants, as it is known that the constitutive expression of genes involved in abiotic stress resistance can hamper the normal growth of transgenic plants (Morran et al. 2011). These results encouraged us to transform a combination of *XvSap*, *XvPrx2* and *XvAld* into two inbred tropical CYMMIT maize lines, respectively, under the control of the *XvSap* promoter. Further work is underway to confirm transformation events prior to subjecting the transformants to water deficit stress.

## Concluding remarks

With predictions that much of sub-Saharan Africa will be desertified by 2060 (Dai 2013), it is important that crops tolerant of extended hot and dry conditions be made available for African farmers. It is our premise that understanding of mechanisms, whereby tolerance of extreme water deficit is achieved will enable informed decisions of what would be required to improve tolerance (rather than just improved resistance characteristics) in annual crops of relevance to such farmers. The resurrection plant *X. viscosa* serves as a model for such understanding. DT is a complex phenomenon and is under the control of numerous interacting factors. In the absence of annotated genome sequence information for this (and any other resurrection plant species), we have used, and describe here, a systems approach in which physiological, biophysical, biochemical and molecular changes that accompany dehydration have been followed. The roles of these in overcoming the main stresses associated with ongoing water deficit (Walters et al. 2002) have been alluded to. While this approach has built a foundation for understanding key changes associated with DT, is has drawbacks, in that subtle but important changes may have been missed. This is due, in part, to the fact that analysis of omic data is reliant on statistical tools that by choice indicate only major changes in tissues at the various RWC analysed. Furthermore, our analysis fails to include nature and roles “orphan” genes/proteins/metabolites noted to change in abundance but the identity of which is as yet unknown. Future studies will include production of a high-quality reference genome for *X. viscosa* and a detailed analysis of tissue-specific transcription factors which regulate changes associated with survival of high levels of water deficit.

In the absence of such information to date, we have relied on data generated by studies reviewed here to select genes for transformation of maize, a staple crop in Africa. While such studies have yielded some evidence of improvement in drought tolerance, as has indeed been reported by other researchers who have attempted to improve this trait in various crops (reviewed in Deikman et al. 2012), they suffer from the drawbacks of trans generational instability and more importantly from the plasticity of a trait which is attained by only small effects generated by each of the genes included. The identification of key tissue-associated regulatory networks controlling DT should ultimately enable manipulated regulation of multiple pathways that are required for tolerance of extreme water loss.

### *Author contribution statement*

JMF initiated research on *X. viscosa,* was the PI of fundamental studies reported and wrote the bulk of the paper. KC compiled tables and figures and contributed to analyses of these data. AH assisted with compilation of molecular data and referencing. KOA conducted nuclear proteome studies. JB wrote the taxonomical aspects of the paper and compiled distribution map. JAT conceived and designed biotechnological studies. HJWD provided and gave input into metabolite data. NP contributed toward interpretation of gene expression data. SGM conceived and designed early transcriptome studies. MSR conceived nuclear proteome studies and was Co-PI of functional analyses of selected protein and gene studies reported. All authors read and edited the paper.

## Abbreviation

ERD (LRD): Early (Late) response to desiccation
